# Chronic Treatment with Multi-Kinase Inhibitors Causes Differential Toxicities on Skeletal and Cardiac Muscles

**DOI:** 10.3390/cancers11040571

**Published:** 2019-04-23

**Authors:** Joshua R. Huot, Alyson L. Essex, Maya Gutierrez, Rafael Barreto, Meijing Wang, David L. Waning, Lilian I. Plotkin, Andrea Bonetto

**Affiliations:** 1 Department of Surgery, Indiana University School of Medicine, Indianapolis, IN 46202, USA; jrhuot@iu.edu (J.R.H.); rafabarreto1@msn.com (R.B.); meiwang@iupui.edu (M.W.); 2 Department of Anatomy and Cell Biology, Indiana University School of Medicine, Indianapolis, IN 46202, USA; alyessex@iu.edu (A.L.E.); lplotkin@iupui.edu (L.I.P.); 3 Greenfield Central High School, Greenfield, IN 46140, USA; m.gutierrez.0928@gmail.com; 4 Department of Cellular and Molecular Physiology, Penn State University, Hershey, PA 17033, USA; dlw83@psu.edu; 5 Department of Otolaryngology—Head & Neck Surgery, Indiana Center for Musculoskeletal Health, Simon Cancer Center, Indiana University School of Medicine, Indianapolis, IN 46202, USA

**Keywords:** chemotherapy, skeletal muscle wasting, cardiac cachexia, sorafenib, regorafenib

## Abstract

Despite recent progress, chemotherapy remains the preferred treatment for cancer. We have shown a link between anticancer drugs and the development of cachexia, i.e., body wasting accompanied by muscle loss. The multi-kinase inhibitors (MKIs) regorafenib and sorafenib, used as second-line treatment for solid tumors, are frequently accompanied by several side effects, including loss of muscle mass and strength. In the present study we aimed to investigate the molecular mechanisms associated with the occurrence of muscle toxicities in in vivo conditions. Hence, we treated 8-week old healthy CD2F1 male mice with MKIs for up to six weeks and observed decreased skeletal and cardiac muscle mass, consistent with muscle weakness. Modulation of ERK1/2 and GSK3β, as well as increased expression of markers of autophagy, previously associated with muscle atrophy conditions, were shown in skeletal muscle upon treatment with either drug. MKIs also promoted cardiac abnormalities consistent with reduced left ventricular mass, internal diameter, posterior wall thickness and stroke volume, despite unchanged overall function. Notably, different signaling pathways were affected in the heart, including reduced expression of mitochondrial proteins, and elevated AKT, GSK3β, mTOR, MEK1/2 and ERK1/2 phosphorylation. Combined, our data demonstrate detrimental effects on skeletal and cardiac muscle in association with chronic administration of MKIs, although different mechanisms would seem to contribute to the cachectic phenotype in the two tissues.

## 1. Introduction

We and others have shown that chemotherapeutic drugs, while effectively combating tumors, can also induce very debilitating side toxicities, including loss of body weight and muscle mass, along with muscle weakness and fatigue [1]. Altogether, these are hallmarks of cachexia, a comorbidity diagnosed in roughly 80% of subjects affected with cancer. Cachexia overall significantly increases the likelihood of disease morbidity and mortality, and will represent the ultimate cause of death for up to 30% of cancer patients [1]. The occurrence of cachexia is ultimately responsible for the onset of a very debilitating state, such that the patients are no longer able to tolerate the anticancer therapies, thus also experiencing discontinuation of treatment and a hastened demise [1]. Along this line, research from our group highlighted the importance of preserving skeletal muscle among subjects receiving chemotherapy treatments, primarily as an essential modality in improving survival rates in cancer, and several studies have demonstrated a correlation between reduced lean muscle mass, dose-limiting toxicities and patient’s survival [2,3,4,5].

According to the most recent statistics, more than 1.7 million new cases of cancer are expected to be diagnosed by the end of 2019 with chemotherapy administration serving as the preferred treatment option [6]. In an attempt to effectively halt tumor progression and metastases, waves of new alternative therapeutics have surfaced in recent years. Among these new classes of drugs are multi-targeted kinase inhibitors (MKIs), two of which, namely sorafenib and regorafenib, have shown significant survival rate improvement in various cancers, including hepatocellular carcinoma, metastatic colorectal cancer, and advanced gastrointestinal stromal tumors [1,7,8]. Despite these promising results, recent studies have identified a myriad of adverse side effects associated with prolonged administration of MKIs, including, but not limited to, arterial hypertension, diarrhea, potential hemorrhage, fatigue and muscle weakness [9,10,11,12,13,14]. Despite early investigations on adverse effects with MKI treatments, the direct molecular impact that these drugs have on skeletal and cardiac muscle is largely unexplored, and studies examining the long-term toxic effects on the musculoskeletal system are lacking.

To better clarify this point we sought to characterize the functional and molecular perturbations of skeletal and cardiac muscle upon chronic administration of sorafenib or regorafenib in normal mice and in the absence of a tumor. Our findings indicate differential deleterious effects of chronic MKI treatment on both muscle types and warrant further investigations into their negative systemic toxicities, especially when administered in combination with other chemotherapeutics.

## 2. Results

### 2.1. Animals Exposed to Regorafenib or Sorafenib Display Impaired Growth

**Figure 1:**
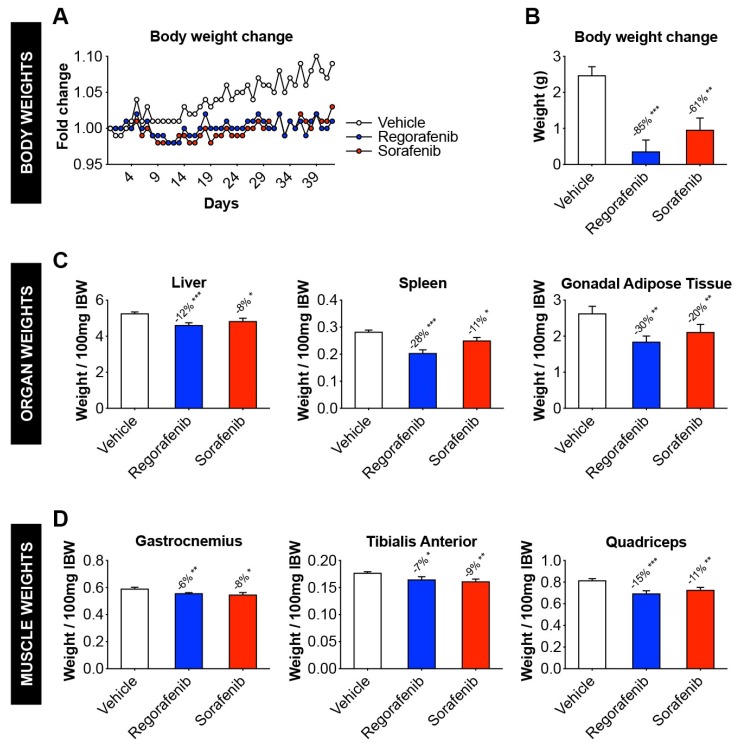
Animals exposed to regorafenib or sorafenib display impaired growth. (**A**) Body weight change (normalized to initial body weight) in mice treated with 30 mg/kg/day regorafenib (blue; *n* = 8), 60 mg/kg/day sorafenib (red; *n* = 8), or vehicle (white; *n* = 8) over the course of 6 weeks. (**B**) Net body weight change (initial to final), expressed in grams. (**C**) Liver, spleen, and gonadal adipose tissue weights (expressed as weight/100 mg Initial Body Weight). (**D**) gastrocnemius, tibialis anterior, and quadriceps muscle weights (expressed as weight/100 mg Initial Body Weight). Data presented as mean ± SEM. Significance of the difference: * *p* < 0.05, ** *p* < 0.01, *** *p* < 0.001 vs. Vehicle.

Animals treated with either regorafenib (30 mg/kg/day) or sorafenib (60 mg/kg/day) for up to six weeks failed to gain weight compared to the vehicle-treated littermates (Figure 1A). Overall, the treated animals displayed a reduction in net weight change compared to vehicle littermates over the course of 6 weeks (regorafenib: −85%, *p* < 0.001; sorafenib: −61%, *p* < 0.01) (Figure 1B). Interestingly, despite a slightly decreased food consumption in the MKI-treated animals, the three experimental groups displayed comparable non-significantly different food intakes (Figure S1). Although the treated mice did not experience dramatic weight loss as typically seen in cachexia, upon normalization to the initial body weight organs such as liver (regorafenib: −12%, *p* < 0.001; sorafenib: −8%, *p* < 0.05), spleen (regorafenib: −28%, *p* < 0.001; sorafenib: −11%, *p* < 0.05), and gonadal adipose tissue (regorafenib: −30%, *p* < 0.01; sorafenib: −20%, *p* < 0.01) showed significant reductions in weight vs. the vehicle-treated animals (Figure 1C). Skeletal muscle weights of gastrocnemius (regorafenib: −6%, *p* < 0.01; sorafenib: −8%, *p* < 0.05), tibialis anterior (regorafenib: −7%, *p* < 0.05; sorafenib: −9%, *p* < 0.01), and quadriceps (regorafenib: −15%, *p* < 0.001; sorafenib: −11%, *p* < 0.01) were significantly less than vehicle littermates, thus suggesting MKIs administration was associated with muscle wasting (Figure 1D).

### 2.2. MKIs Promote Skeletal Muscle Weakness

**Figure 2:**
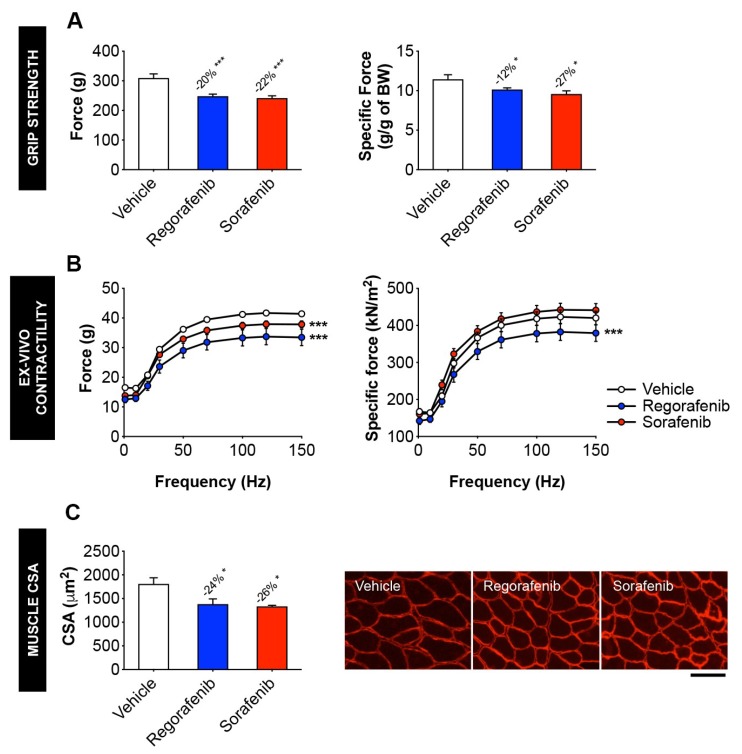
MKIs promote skeletal muscle weakness. (**A**) Assessment of grip strength, reported as absolute force (expressed in grams) or specific force (expressed relative to body weight (BW)) in mice treated with 30 mg/kg/day regorafenib (blue; *n* = 8), 60 mg/kg/day sorafenib (red; *n* = 8), or vehicle (white; *n* = 8) over the course of 6 weeks. (**B**) Assessment of whole muscle contractility of EDL muscle, reported as absolute muscle force (expressed in grams) and specific force (expressed as kN/m^2^). (**C**) Cross-sectional area (CSA) of tibialis anterior muscles and representative CSA image of tibialis anterior muscle sections stained with anti-dystrophin antibody. Images taken at 20×, scale bar equals 100 µm. Data presented as mean ± SEM. Significance of the difference: * *p* < 0.05, *** *p* < 0.001 vs. Vehicle.

In vivo grip strength measurement revealed concurrent decreases in absolute (regorafenib: −20%, *p* < 0.001; sorafenib: −22%, *p* < 0.001) and specific (regorafenib: −12%, *p* < 0.05; sorafenib: −27%, *p* < 0.05) force for animals treated with either compound (Figure 2A). Analogously, whole muscle contractility testing of the EDL muscles revealed similar effects in muscle contractility, with regorafenib reducing both absolute (*p* < 0.001) and specific force (*p* < 0.001), and sorafenib lowering absolute force (*p* < 0.001) when compared to the control animals (Figure 2B). Consistent with decreases in weight and strength, myofibers from MKI-treated animals were significantly smaller than in the vehicle-treated littermates, as suggested by the assessment of cross-sectional area (CSA) (Figure 2C).

### 2.3. Regorafenib and Sorafenib Perturb Cardiac Muscle

**Figure 3:**
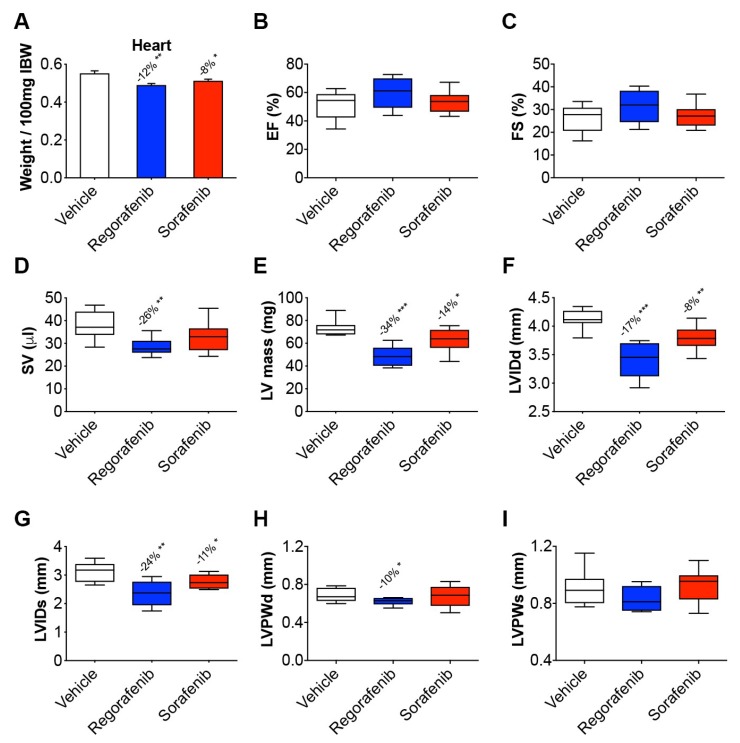
Regorafenib and sorafenib perturb cardiac muscle. Cardiac function measured by echocardiography in mice treated with 30 mg/kg/day regorafenib (blue; *n* = 8), 60 mg/kg/day sorafenib (red; *n* = 8), or vehicle (white; *n* = 8) over the course of 6 weeks. (**A**) Heart size (relative to initial body weight). (**B**) Ejection fraction (EF). (**C**) Fractional shortening (FS). (**D**) Stroke volume (SV). (**E**) Left ventricular (LV) mass. (**F**) Left ventricular inner wall diameter (LVID) during diastole. (**G**) LVID during systole. (**H**) Left ventricular posterior wall (LVPW) thickness during diastole. (**I**) LVPW thickness during systole. Data presented as mean ± SEM. Significance of the difference: * *p* < 0.05, ** *p* < 0.01, *** *p* < 0.001 vs. Vehicle.

Skeletal muscle dysfunction was also accompanied by deleterious modifications to cardiac function in MKI treated animals. Indeed, heart weight was significantly reduced in the animals receiving either regorafenib (−12%, *p* < 0.01) or sorafenib (−8%, *p* < 0.05), thereby suggesting cardiac toxicity in response to treatment with MKIs (Figure 3A). Using echocardiography, ejection fraction (Figure 3B) and fractional shortening (Figure 3C) were found unchanged in the treated animals, although significant reductions in stroke volume (regorafenib: −26%, *p* < 0.01) (Figure 3D), left ventricular mass (regorafenib: −34%, *p* < 0.001; sorafenib: −14%, *p* < 0.05) (Figure 3E) and left ventricular inner wall diameter (LVID) during both diastole (regorafenib: −17%, *p* < 0.001; sorafenib: −8%, *p* < 0.01) (Figure 3F) and systole (regorafenib: −24%, *p* < 0.01; sorafenib: −11%, *p* < 0.05) (Figure 3G) were detected. Moreover, left ventricular posterior wall (LVPW) thickness was comparable to the control animals, with the exception of LVPW during diastole in regorafenib treated animals (−10%, *p* < 0.05) (Figure 3H,I).

### 2.4. MKIs Affect Cachexia-Related Pathways in Skeletal Muscle

**Figure 4:**
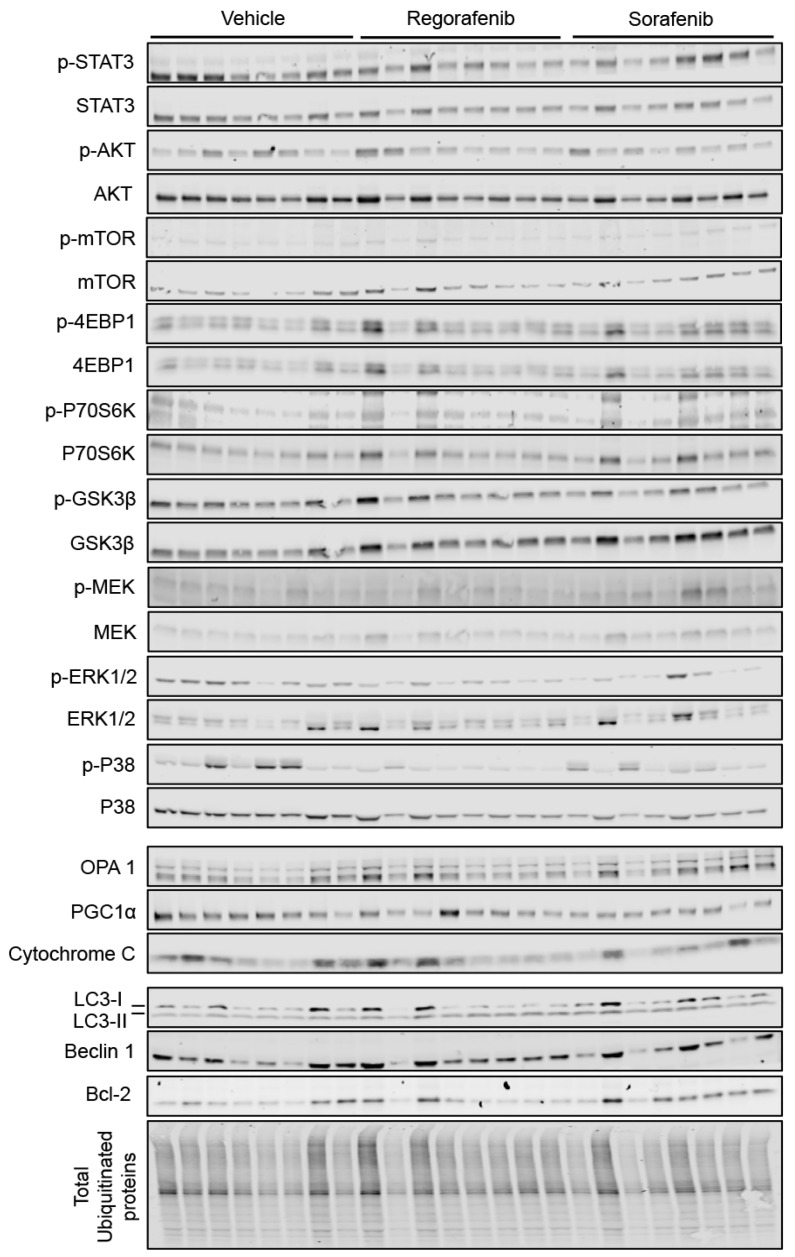

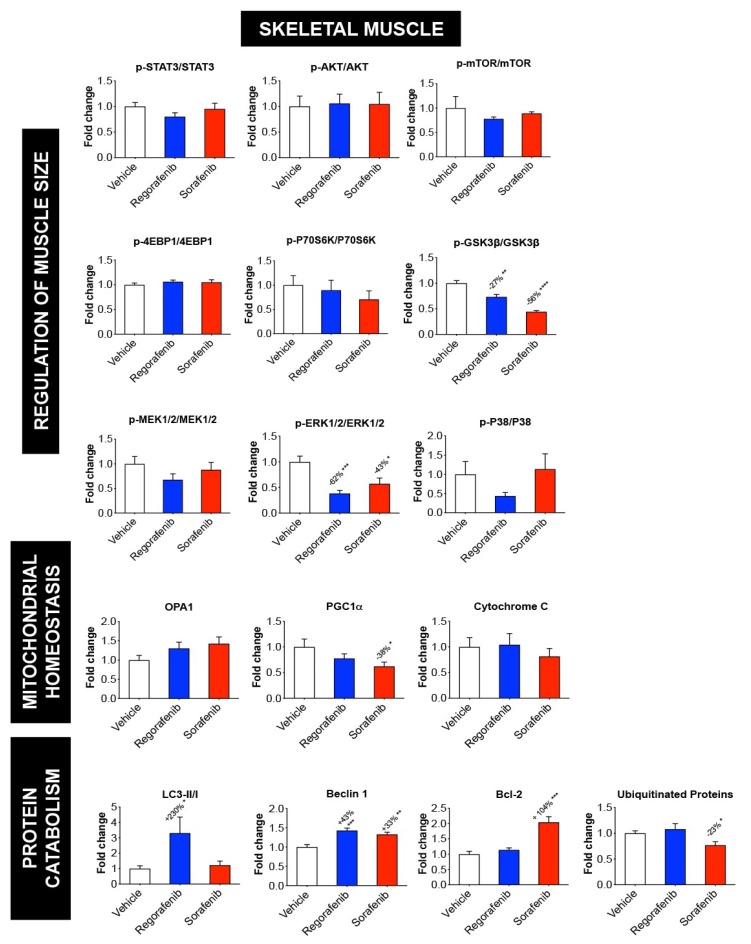
MKIs determine skeletal muscle atrophy. Representative western blotting and quantification (expressed as fold change vs. Vehicle) of proteins involved in the regulation of muscle size (STAT3, AKT, mTOR, 4EBP1, P70S6K, GSK3β, P38, MEK1/2, ERK1/2) (Top), proteins involved in mitochondrial homeostasis (OPA1, PGC1α, Cytochrome C) (Middle), and protein markers of autophagy-dependent catabolism (LC3, Beclin 1, Bcl-2 and total ubiquitinated proteins) (Bottom) in whole skeletal muscle protein extracts from mice treated with 30 mg/kg/day regorafenib (blue; *n* = 8), 60 mg/kg/day sorafenib (red; *n* = 8), or vehicle (white; *n* = 8) over the course of 6 weeks. Levels of phosphorylated proteins were normalized to their respective total protein. Tubulin served as the loading control. Data presented as mean ± SEM. Significance of the difference: * *p* < 0.05, ** *p* < 0.01, *** *p* < 0.001, **** *p* < 0.0001 vs. Vehicle.

In order to gain molecular insight into the pro-cachectic symptoms observed with regorafenib and sorafenib administration, we analyzed the expression of proteins previously characterized in association with muscle atrophy in cachexia due to cancer and/or chemotherapy. Neither MKI affected the activation of catabolic or anabolic signaling proteins, including STAT3, AKT, mTOR, or the downstream mTOR effectors 4EBP1 and P70S6K (Figure 4). Interestingly, GSK3β was significantly down-regulated (regorafenib: −27%, *p* < 0.01; sorafenib: −56%, *p* < 0.0001). On the other hand, regorafenib and sorafenib treatment significantly down-regulated ERK1/2 (regorafenib: −62%, *p* < 0.001; sorafenib: −43%, *p* < 0.05), despite no changes in P38 or the upstream ERK1/2 regulator, MEK1/2. (Figure 4). Additionally, neither regorafenib nor sorafenib altered the expression of mitochondrial proteins that we previously linked to skeletal muscle wasting, including OPA1 or cytochrome C [2], whereas only PGC1α expression was reduced with sorafenib treatment (−38%, *p* < 0.05) (Figure 4). These findings suggest little effect of MKIs administration on muscle metabolism and ox-phos potential.

**Figure 5:**
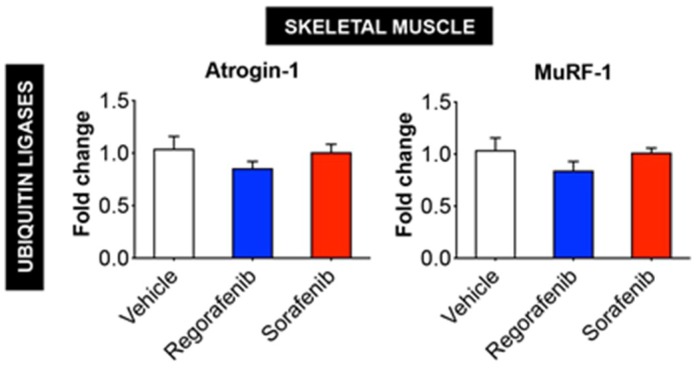
Expression of ubiquitin ligases Atrogin-1 and MuRF-1 in skeletal muscle is not affected by MKIs. mRNA expression for Atrogin-1 and MuRF-1 in the skeletal muscle of mice treated with 30 mg/kg/day regorafenib (blue; *n* = 8), 60 mg/kg/day sorafenib (red; *n* = 8), or vehicle (white; *n* = 8) over the course of 6 weeks. Data presented as mean ± SEM.

Alternatively, LC3-II/I (regorafenib: +230%, *p* < 0.05), Beclin 1 (regorafenib: +43%, *p* < 0.001; sorafenib: +33%, *p* < 0.01) and Bcl-2 (sorafenib: +104%, *p* < 0.001) levels were significantly increased in animals receiving MKIs, thereby suggesting that treatment with these chemotherapeutics elevates protein markers of autophagy-dependent muscle catabolism. On the other hand, the amount of total ubiquitinated protein was reduced upon sorafenib treatment alone (−23%, *p* < 0.05; Figure 4), whereas no significant change was observed in the expression of the ubiquitin ligases Atrogin-1 and MuRF-1 (Figure 5). Taken together, these data support the idea that regorafenib and sorafenib play a causative role in skeletal muscle atrophy primarily by promoting enhanced autophagy-dependent catabolism.

### 2.5. Regorafenib and Sorafenib Alter Cachexia-Associated Pathways in Cardiac Muscle

**Figure 6:**
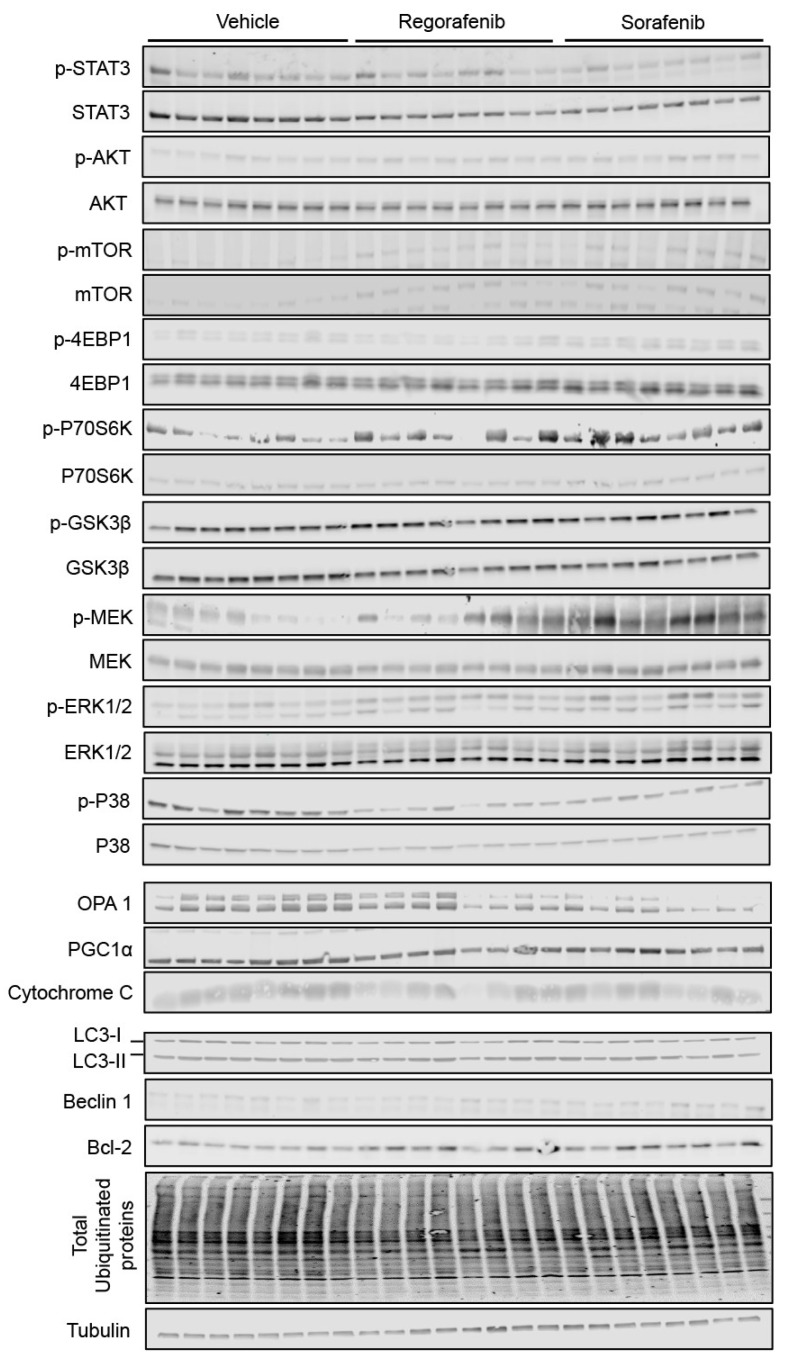

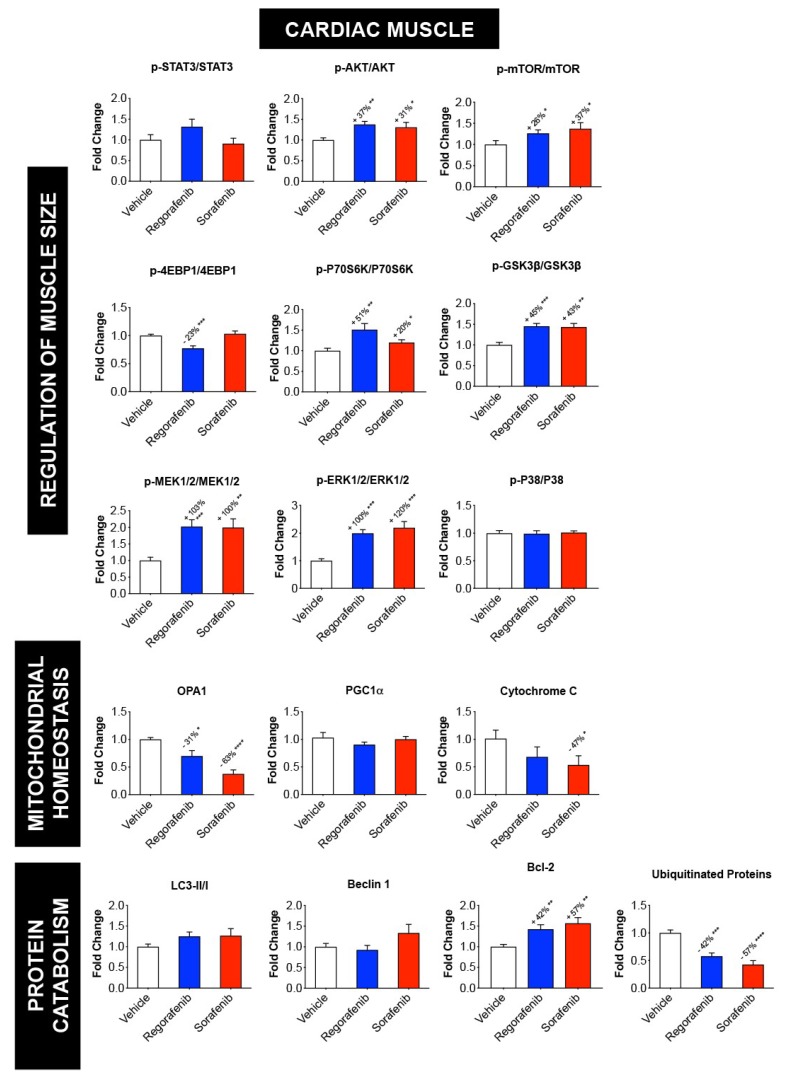
Regorafenib and sorafenib alter cachexia-associated pathways in cardiac muscle. Representative western blotting and quantification (expressed as fold change *vs.* Vehicle) of proteins involved in the regulation of muscle size (STAT3, AKT, mTOR, 4EBP1, P70S6K, GSK3β, P38, MEK1/2, ERK1/2) (Top), proteins involved in mitochondrial homeostasis (OPA1, PGC1α, Cytochrome C) (Middle), and markers of protein catabolism, (LC3, Beclin 1, Bcl-2 and total ubiquitinated proteins) (Bottom) in whole cardiac muscle protein extracts from mice treated with 30 mg/kg/day regorafenib (blue; *n* = 8), 60 mg/kg/day sorafenib (red; *n* = 8), or vehicle (white; *n* = 8) over the course of 6 weeks. Levels of phosphorylated proteins were normalized to their respective total protein. Tubulin served as the loading control. Data presented as mean ± SEM. Significance of the difference: * *p* < 0.05, ** *p* < 0.01, *** *p* < 0.001, **** *p* < 0.0001 vs. Vehicle.

Unlike was observed in skeletal muscle, phosphorylation of AKT was enhanced by both regorafenib (+37%, *p* < 0.01) and sorafenib (+31%. *p* < 0.05); consistently, both MKIs increased the phosphorylation of downstream AKT mediators, including mTOR (regorafenib: +26%, *p* < 0.05; sorafenib: +37%, *p* < 0.05), P70S6K (regorafenib: +51%, *p* < 0.01; sorafenib: +20%, *p* < 0.05) and GSK3β (regorafenib: +45%, *p* < 0.001; sorafenib: +43%, *p* <0.01), while 4EBP1 was decreased following regorafenib treatment (−23%, *p* < 0.001) (Figure 6). Interestingly, both MKIs induced marked activation of MEK1/2 (regorafenib: +103%, *p* < 0.001; sorafenib: +100%, *p* < 0.01) and of the downstream target ERK1/2 (regorafenib: +100%, *p* < 0.001; sorafenib: +120%, *p* < 0.001). Similar to the skeletal muscle, activation of P38 and STAT3 was unchanged upon MKI treatment (Figure 6).

**Figure 7:**
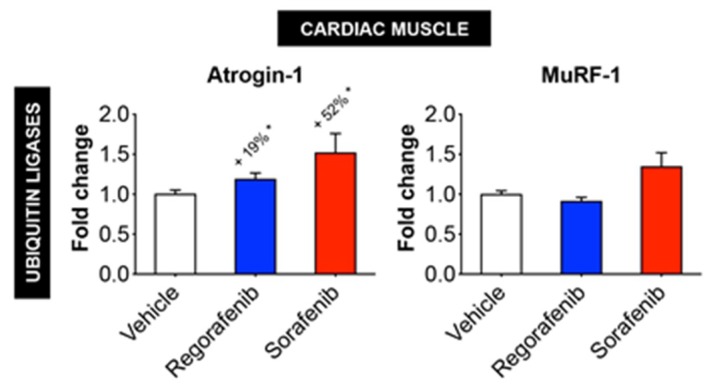
MKIs determine increased expression of cardiac Atrogin-1, whereas MuRF-1 is unchanged. mRNA expression for Atrogin-1 and MuRF-1 in the cardiac muscle of mice treated with 30 mg/kg/day regorafenib (blue; *n* = 8), 60 mg/kg/day sorafenib (red; *n* = 8), or vehicle (white; *n* = 8) over the course of 6 weeks. Data presented as mean ± SEM. Significance of the difference: * *p* < 0.05 vs. Vehicle.

Also differing from skeletal muscle, the mitochondrial proteins OPA1 (regorafenib: −31%, *p* < 0.05; sorafenib: −63%, *p* < 0.001) and cytochrome C (sorafenib: −47%; *p* < 0.05) were significantly reduced (Figure 6), while neither LC3 nor Beclin 1 were affected by MKIs (Figure 6). On the other hand, Bcl-2 was significantly increased (regorafenib: +42%, *p* < 0.01; sorafenib: +57%, *p* < 0.01), whereas the amount of total ubiquitinated proteins was reduced upon treatment with both regorafenib (−42%, *p* < 0.001) and sorafenib (−57%, *p* < 0.0001) (Figure 6). Interestingly, both drugs determined increased expression of Atrogin-1 (regorafenib: +19%, *p* < 0.05; sorafenib: +52%, *p* < 0.05), whereas MuRF-1 was unchanged (Figure 7). Furthermore, expression of BNP, a marker of cardiac hypertrophy [15,16], was also elevated in the heart of animals exposed to regorafenib (+33%, *p* < 0.001) and sorafenib (+25%), although in the latter case the difference did not reach statistical significance (Figure S2). Altogether, these findings identified distinct differences in cardiac muscle compared to skeletal muscle.

## 3. Discussion

Novel second line chemotherapeutics, including the MKIs regorafenib and sorafenib, have been developed over the past decade for the treatment of advanced and metastatic solid tumors. Regorafenib, an inhibitor of VEGFR, was developed to counteract the angiogenic activity in several solid cancers, including metastatic colorectal cancer and advanced gastrointestinal tumors [17]. On the other hand, sorafenib, initially designed to inhibit b-Raf, VEGFR and PDGFR, was shown to effectively prolong survival among patients affected with metastatic hepatocellular carcinoma [8,18,19,20,21,22]. Despite their potent anti-proliferative effects, several toxicities frequently accompany MKI administration, and whether prolonged administration of such compounds promotes side effects per se, including muscle loss and weakness, is yet to be determined.

In order to clarify this point we exposed normal, healthy mice to doses of chemotherapeutics that had been previously described in the literature as effective in counteracting tumor growth in rodent models, and that, due to the prolonged time of administration, were also likely to cause side effects [18,23]. Interestingly, based on the 2005 USFDA guide for the dose conversion between animals and humans (as also discussed in [24]), the dosing used in our study was comparable to the ones generally prescribed to subjects with cancer in the clinical setting (e.g., 160 mg for regorafenib or 800 mg for sorafenib, daily, for subjects weighing about 60 kg). To the extent of investigating the causative mechanisms for the occurrence of defects in skeletal and cardiac muscles, the animals were sacrificed when a condition of mild-to-severe cachexia had become evident, as we previously described in models of cancer- and chemotherapy-induced cachexia [3,25]. In this case, the animals were exposed to the MKIs for up to six weeks in order to allow the appearance of toxicities associated with administration of such drugs. This is also in line with observations reported in a phase II study aimed at evaluating tolerability and efficacy of sorafenib in patients with refractory cancers, showing that the drug was administered for an average of 68 days and up to 344 days [26].

The present findings indicate that both regorafenib and sorafenib negatively affect growth and promote skeletal and cardiac muscle wasting in normal mice. Ours is not the first evidence suggesting a role of MKIs in causing muscle-associated deficits. Indeed, regorafenib was recently shown to worsen the survival outcomes in refractory metastatic colorectal cancer patients, in particular in association with low skeletal muscle mass [27,28,29]. Along the same line, sorafenib administration in experimental animals was reported to cause body weight loss [18], and Antoun et al. concluded that sorafenib was likely to cause muscle wasting in patients affected with advanced renal cell carcinoma due to the specificity for the Raf kinase, normally involved in the regulation of muscle mass [19]. Similar to other multi-targeted kinase inhibitors, such as imatinib and sunitinib, sorafenib treatment was also shown to cause abnormal mitochondrial functions and, in turn, alterations of the energy metabolism, likely responsible for muscle weakness [30]. Interestingly, other studies failed to demonstrate changes in body weight in tumor hosts exposed to regorafenib [31] or sorafenib [32], although in both cases only tumor-bearing mice were tested.

Here we did not report severe decline in body weight over the course of the 6 weeks of treatment. Instead, significant retardation in growth with both sorafenib and regorafenib treatment was evident with respect to the vehicle-treated animals. Although the growth retardation observed may represent a limitation of our study and is likely attributable to the fact that younger animals (i.e., 8 weeks of age) were administered MKIs, compared to previous studies which utilized older weight-stable mice (i.e., 14–16 weeks of age) [33], this subject matter remains of particular interest and may warn against administration of such agents in pediatric cancer patients. This is especially true considering that other multi-target kinase inhibitors, including imatinib, were previously shown to cause growth failure in children affected with chronic myeloid leukemia primarily by perturbing the GH:IGF-1 axis [34].

In our study we also detected a significant decrease in heart weight after six weeks of treatment with regorafenib or sorafenib. This, combined with the increased expression of the ubiquitin ligase Atrogin-1, generally indicative of enhanced protein catabolism [35], and with the reduction in left ventricular mass and inner wall diameter, is supportive of a phenotype consistent with cardiac cachexia in the mice receiving MKIs. Our results are in line with previous findings showing significant reductions in heart size in 12-week old mice treated with sorafenib in conjunction with myocardial infarction [36]. Notably, in our experimental model alterations in cardiac phenotype (e.g., reduced heart size, LV mass, LVID) were not accompanied by reductions in ejection fraction or fractional shortening with either MKI, in line with the previous study from Duran et al. conducted in animals exposed to sorafenib [31]. In contrast, earlier observations demonstrated significant decrease in cardiac function with two weeks of sorafenib treatment [37]. These different outcomes could be attributed to the different time point taken into consideration (2 weeks vs. 6 weeks), and to the fact that isoflurane-induced anesthesia was not applied at time of measurement. Indeed, Pachon et al. demonstrated that the use of anesthetics might suppress changes in both ejection fraction and fractional shortening [38].

Despite the knowledge that other chemotherapeutics induce cardiac and skeletal muscle perturbations, studies delving into distinct molecular signatures in skeletal and cardiac muscles that may be contributing to the occurrence of muscle atrophy with regorafenib and sorafenib are scarce. Our study has highlighted differential molecular alterations that occur in skeletal and cardiac muscle relative to signaling pathways that have been previously implicated in regulating muscle growth and that have been linked to the occurrence of a cachectic muscle phenotype.

The most striking differences were found in the activation of ERK1/2 MAPK. In particular, while ERK1/2 phosphorylation was reduced in the skeletal muscle of animals receiving regorafenib or sorafenib, we showed opposite trends in the cardiac muscle. Interestingly, although still being debated, activation of the signaling dependent on MEK1/2 and ERK1/2 in the heart seems to be directly linked with hypertrophy, as also elegantly reviewed in [39]. This may represent a discrepancy with the phenotype described in the mice receiving MKI treatment, resulting in cardiac atrophy. However, despite the fact that the animals presented smaller hearts, we did not see any change in cardiac function. Altogether, these observations may suggest that compensatory mechanisms take place after chronic administration with MKIs, as also suggested by the activation of signaling pathways normally associated with the growth of the cardiac muscle (such ERK1/2 and AKT/mTOR/P70S6K). Our speculations are further supported by the fact that expression of BNP, a known marker of cardiac hypertrophy [15,16], was significantly upregulated in the hearts of mice chronically exposed to MKIs. On the other hand, ours is not the first evidence that anticancer drugs can cause cardiac toxicity accompanied by ERK1/2 activation, as also recently showed in [40].

Similarly, here we demonstrated that neither regorafenib nor sorafenib determined changes in the phosphorylation of AKT in skeletal muscle, despite reduced ser9 phosphorylation of GSK3β. Interestingly, ERK1/2 has also been implicated in the regulation of GSK3β, in that it specifically associates with and primes GSK3β for deactivation via ser9 phosphorylation [41,42]. It is plausible that skeletal muscle changes in GSK3β with MKI administration are contingent on ERK 1/2 activity as opposed to AKT. On the other hand, within cardiac muscle both MKIs promoted elevated phospho-AKT levels as well as increased GSK3β phosphorylation. Similarly (yet opposite to skeletal muscle), increased ERK1/2 phosphorylation is associated with increased GSK3β phosphorylation further suggesting that, at least in the case of MKI treatments, this kinase might rather be regulated by ERK1/2, similar to previous observations generated in animals exposed to radiations [41].

Interestingly, the expression of markers of the ATP-ubiquitin-dependent protein degradation (e.g., ubiquitin ligases Atrogin-1 and MuRF-1 and protein ubiquitination), typically altered in cachexia [43], were only modestly affected by MKI treatments, thus suggesting that other mechanisms were likely involved in promoting skeletal muscle wasting. Along this line, regorafenib and sorafenib are known to elevate autophagic markers, including Beclin1 and LC3, within various tumor cells [38], although the systemic autophagic impact of these MKIs has yet to be reported in muscle. Here we demonstrated that both sorafenib and regorafenib induced elevations in Beclin 1 and LC3-II/I in skeletal muscle, thus supporting the idea that autophagy activation may trigger muscle depletion in our experimental model, similar to previous findings in a cancer cachexia setting [44]. Conversely, no changes were detected in cardiac tissue. Interestingly, Bcl-2, an anti-apoptotic protein and negative regulator of autophagy by means of its direct interaction with Beclin 1 [45], was markedly upregulated following sorafenib treatment in both skeletal and cardiac muscles, and only in the heart upon regorafenib administration. Notably, similar increases in Bcl-2 levels were recently reported in a model of cardiotoxin-induced muscle injury [46]. Altogether, our findings may suggest an attempt to inhibit autophagy in order to preserve muscle mass.

In the present study we also showed that mitochondrial proteins, such as OPA1 and cytochrome C, that we previously reported down-regulated in models of cancer- or chemotherapy-induced muscle wasting [2,43], were reduced in cardiac muscle with MKI treatment, whereas these same proteins were unchanged in skeletal muscle, also in line with previously published evidence investigating the mitochondrial toxicity induced by sorafenib [31,47] or supporting the MKI-induced disruption of mitochondrial membrane polarization in tumor cell lines [48].

Lastly, here we demonstrated that sorafenib and regorafenib do not alter STAT3 phosphorylation in either skeletal or cardiac muscle, despite the fact that minimal changes in phospho-STAT3 were previously observed in the heart following administration of sorafenib [49,50,51,52] or in tumor hosts [53]. Interestingly, Toledo et al. previously showed that daily treatment with sorafenib (90 mg/kg per os) was able to counteract cancer-induced muscle protein depletion in the C26 mouse model [36]. Notably, the authors claimed sorafenib negatively regulates the activation of STAT3, a transcription factor that we reported to be markedly elevated in the muscle of tumor hosts and directly involved in the pathogenesis of cancer-associated cachexia [25], thus also partially explaining the beneficial effects observed in tumor hosts. Altogether, such observations may initially appear in disagreement with our study, showing, on the contrary, loss of muscle mass following administration of sorafenib, despite the absence of changes in STAT3 phosphorylation in either skeletal or cardiac muscles. However, since in the study by Toledo et al. [36] the sorafenib-related effects were tested exclusively in animals bearing solid tumors and for shorter periods (up to 2 weeks), at this time we are prevented from performing a direct comparison with the model described in our study. Regardless, we could speculate that the beneficial effects associated with STAT3 blockade in the muscle of animals bearing highly inflammatory tumors (such the C26 colorectal adenocarcinomas) are likely to overcome the toxicities of the drug per se.

## 4. Materials and Methods

### 4.1. Animals

All experiments were conducted with the approval of the Institutional Animal Care and Use Committee at Indiana University School of Medicine (Animal Welfare Assurance n. D16-00584, Protocol n. 10759MD/R/E, approved on 13 August 2014) and were in compliance with the National Institutes of Health Guidelines for Use and care of Laboratory Animals. The animals were acclimated for at least one week upon delivery and before any manipulation. Eight-week-old CD2F1 male mice (*n* = 8; Envigo, Indianapolis, IN, USA) were maintained on a Teklad Global Rodent Diet (#2018X; Madison, WI, USA) and were administered per os (daily, by gavage regorafenib (30 mg/kg/day; Selleck Chemicals #S1178, Houston, TX, USA) or sorafenib (60 mg/kg/day; Selleck Chemicals #S7397) dissolved in Cremophor EL/ethanol (50:50) and diluted in sterile water over the course of six weeks [18]. Control mice received an equal volume of vehicle. Mice were weighed daily. Food intake was measured on a daily basis by weighing the amount of food consumed for each cage. On day 42, the animals were euthanized under light isoflurane anesthesia. Tissues were collected, weighed, and snap frozen in liquid nitrogen and stored at −80 °C for further analysis. Tibialis anterior muscles were frozen in liquid nitrogen-cooled isopentane, mounted in OCT and stored at −80 °C for morphological analyses, as shown in [54].

### 4.2. Grip Strength Measurement

Forelimb strength was assessed using a commercially available automatic grip strength meter (Columbus Instruments, Columbus, OH, USA), as previously described [55]. The absolute force (expressed in grams) and the normalized force (expressed as grams of force/body weight) were recorded. To reduce procedure related variability, the same operator analyzed an average from several repeated peak force measurements in the same animal in a blind manner. For this assay, five measurements were performed, and the top three measurements were used for the analysis. Moreover, to avoid bias of habituation, the animals were tested once a week during the experimental period.

### 4.3. Muscle Cross-Sectional Area (CSA)

Ten μm-thick cryosections of tibialis anterior muscles taken at the mid-belly were processed for immunostaining. Samples were marked with a PAP pen, blocked in phosphate buffered saline (PBS) containing 8% bovine serum albumin for one hour at room temperature, and incubated at 4 °C overnight with dystrophin primary antibody (Developmental Studies Hybridoma Bank, Iowa City, IA, USA; #MANDRA1(7A10)) diluted in PBS. After the overnight incubation, samples were incubated with a secondary antibody (AlexaFluor 594 # A-11032; ThermoFisher Scientific, Waltham, MA, USA) for one hour. Samples were then washed with PBS, mounted, and imaged using an Axio Observer.Z1 motorized microscope (Zeiss, Oberchoken, Germany). For determination of the CSA, muscle fibers (*n*  =  300–500 per sample) were measured by tracing the perimeter of each individual fiber using a Cintiq pen tablet input device (Wacom, Vancouver, WA, USA) and Image J 1.43 software [56,57].

### 4.4. Whole Muscle Contractility

Whole muscle contractility of the extensor digitorum longus (EDL) muscles was determined as previously described [58]. EDLs were dissected from hind limbs; stainless steel hooks were tied to the tendons of the muscles using 4–0 silk sutures, and the muscles were mounted between a force transducer (Aurora Scientific, Aurora, ON, Canada) and an adjustable hook. The muscles were immersed in a stimulation chamber containing O_2_/CO_2_ (95/5%) and bubbled Tyrode solution (121 mM NaCl, 5.0 mM KCl, 1.8 mM CaCl2, 0.5 mM MgCl_2_, 0.4 mM NaH_2_PO_4_, 24 mM NaHCO_3_, 0.1 mM EDTA, 5.5 mM glucose). The muscle was stimulated to contract using a supramaximal stimulus between two platinum electrodes. Data was collected via Dynamic Muscle Control/Data Acquisition (DMC) and Dynamic Muscle Control Data Analysis (DMA) programs (Aurora Scientific). At the start of each experiment the muscle length was adjusted to yield the maximum force. The force–frequency relationships were determined by triggering contraction using incremental stimulation frequencies (0.5 ms pulses at 1–150 Hz for 350 ms at supramaximal voltage). Between stimulations, the muscle was allowed to rest for 3 min. At the end of the force measurement, the length (L0) and weight of the muscle was measured to facilitate determination of the specific force. Specific force is the absolute force normalized to the muscle the cross-sectional area, calculated as shown in [59]. The investigators were blinded to the treatment of subjects.

### 4.5. Echocardiography

The potential cardiac influence of MKIs was determined in regorafenib- or sorafenib-treated mice via echocardiography using the Vevo^®^ 2100 system (Fujifilm VisualSonics Inc., Toronto, ON, Canada). Six weeks after daily injections of MKIs, mice were placed under isoflurane anesthesia for assessment of cardiac function and muscle mass alteration with a heart rate maintained at 400–500 beats per minute. M-mode scanning of the left ventricular chamber was used for analysis of left ventricular (LV) ejection fraction (EF), fractional shortening (FS), stroke volume (SV), LV internal diameter (diastole/systole) (LVIDd/s) and LV posterior wall thickness (diastole/systole) (LVPWd/s).

### 4.6. Western Blotting

Total protein extracts were obtained by homogenizing 50 mg quadriceps muscle tissue or whole-heart tissue in RIPA buffer (150 mM NaCl, 1.0% NP-40, 0.5% sodium deoxycholate, 0.1% SDS, and 50 mM Tris, pH 8.0) completed with inhibitor cocktails for proteases (Roche, Indianapolis, IN, USA) and phosphatases (Thermo Scientific, Rockford, IL, USA). Cell debris were removed by centrifugation (15 min, 14,000× *g*) and the supernatant collected and stored at −80 °C. Protein concentration was determined using the BCA protein assay method (Thermo Scientific). Protein extracts (30 μg) were then electrophoresed in 4–15% gradient SDS Criterion TGX precast gels (Bio-Rad, Hercules, CA, USA). Gels were transferred to nitrocellulose membranes (Bio-Rad, Hercules, CA, USA). Membranes were blocked with SEA BLOCK blocking reagent (Thermo Scientific) at room temperature for 1 h, followed by an overnight incubation with diluted antibody in SEA BLOCK buffer (Thermo Scientific) containing 0.2% Tween-20 at 4 °C with gentle shaking. After washing with PBS containing 0.2% Tween-20 (PBST), the membrane was incubated at room temperature for 1 h with either anti-rabbit IgG (H+L) DyLight 800 or anti-mouse IgG (H+L) DyLight 680 secondary antibodies (Cell Signaling Technologies, Danvers, MA, USA). Blots were then visualized with Odyssey Infrared Imaging System (LI-COR Biosciences, Lincoln, NE, USA). Optical density measurements were taken using the Gel-Pro Analyzer software. Antibodies used were phospho-MEK1/2 (Ser 217/221) (#9154), MEK1/2 (#9126), phospho-ERK1/2 (Thr202/Tyr204) (#4370), ERK1/2 (#4695), phospho-p38 (Thr180/Tyr182) (#4511), p38 (#9212), phospho-AKT (Ser473) (#4060), AKT (#9272), phospho-mTOR (Ser2448) (#5536), mTOR (#2983), phospho-p70S6K (Thr389) (#9234), p70S6K (9209), phospho-4EBP1 (Thr37/46) (#2855), 4EBP1 (#9644), phospho-GSK-3β (Ser9) (#5558), GSK-3β (#12456), OPA-1 (#80471), Cytochrome C (#11940), phospho-STAT3 (Tyr705) (#9145), STAT3 (#12640), Ubiquitin (#3933) from Cell Signaling Technologies, Beclin1 (#B6186), LC3 (#L7543), PGC-1α (#AB3242) from MilliporeSigma (Burlington, MA, USA), anti-Bcl-2 (#ab182858) from Abcam (Cambridge, MA, USA) and α-Tubulin (#12G10) from Developmental Studies Hybridoma Bank (Iowa City, IA, USA). In general, phosphorylated protein levels were normalized to the expression of the respective total proteins. LC3 was presented as ratio between LC3-II and LC3-I. Tubulin was used as loading control.

### 4.7. Real-Time Quantitative Polymerase Chain Reaction (qRT-PCR)

RNA from quadriceps and heart was isolated using the miRNeasy Mini kit (Qiagen, Valencia, CA, USA) and following the protocol provided by the manufacturer. RNA was quantified using a Synergy H1 spectrophotometer (BioTek, Winooski, VT, USA). RNA integrity was checked by electrophoresis on a 1.2% agarose gel containing 0.02 mol/L morpholinopropanesulfonic acid and 18% formaldehyde. Total RNA was reverse transcribed to cDNA using the Verso cDNA kit (Thermo Fisher Scientific, Waltham, MA, USA). Transcript levels were measured by Real-Time PCR (Light Cycler 96, Roche, Indianapolis, IN, USA) taking advantage of the TaqMan gene expression assay system (Life Technologies, Carlsbad, CA, USA). Expression levels for Atrogin-1 (Mm00499523_m1), MuRF-1 (Mm01185221_m1) and Natriuretic Peptide B (BNP; Mm01255770_g1) were detected. Gene expression was normalized to TBP (Mm01277042_m1) levels using the standard 2^−ΔΔCT^ methods.

### 4.8. Statistics

Results are presented as means ± SEM. Significance of the differences was determined by analysis of variance (ANOVA) followed by Tukey’s post-test. Differences were considered significant when *p* < 0.05.

## 5. Conclusions

In conclusion, here we presented evidence that MKI administration promotes growth retardation and negatively impact skeletal and cardiac muscles, leading to atrophy and loss of function, which are accompanied by negative molecular alterations, including increased levels of autophagy-dependent protein markers and abnormal mitochondrial homeostasis. Given the well-described role of skeletal and cardiac muscles in promoting better outcomes and longer survival rates in patients with cancer, further investigation into the negative systemic effects of sorafenib and regorafenib, particularly in combination with other routinely used chemotherapeutics, is warranted.

## Supplementary Materials

The following are available online at https://www.mdpi.com/2072-6694/11/4/571/s1, Figure S1: Food consumption is not affected by regorafenib or sorafenib, Figure S2: BNP mRNA expression is increased in animals treated with MKIs.

## Abbreviations

MKI	multi-kinase inhibitor
ERK1/2	extracellular signal-regulated kinase 1/2
STAT3	signal transducer and activator of transcription 3
GSK3b	glycogen synthase kinase-3 beta
AKT	protein kinase b
mTOR	mechanistic target of rapamycin
p70S6K	70 kDa ribosomal protein S6 kinase
4EBP1	eukaryotic initiation factor 4E binding protein
MEK1/2	mitogen-activated protein kinase kinase
OPA1	optic atrophy protein 1
PGC1a	peroxisome proliferator-activated receptor gamma co-activator 1 alpha
LC3	microtubule-associated protein 1A/1B-light chain 3
Bcl-2	b-cell lymphoma 2
BNP	brain natriuretic peptide
TBP	TATA-binding protein
CSA	cross-sectional area
SV	stroke volume
LV	left ventricle
LVIDd	left ventricular internal diameter during diastole
LVIDs	left ventricular internal diameter during systole
LVPWd	left ventricular posterior wall thickness during diastole
LVPWs	left ventricular posterior wall thickness during systole
