# Supplementary material for: Chronic Treatment with Multi-Kinase Inhibitors Causes Differential Toxicities on Skeletal and Cardiac Muscles
Source: Cancers (Basel). 2019 Apr 23;11(4):571. doi: 10.3390/cancers11040571 (PMC6520777; doi:10.3390/cancers11040571)
Supplement: Supplementary file 1 [file cancers-11-00571-s001.pdf]

Supplementary Materials: Chronic Treatment with Multi-Kinase Inhibitors Causes Differential Toxicities on Skeletal and Cardiac Muscles

Joshua R. Huot, Alyson L. Essex, Maya Gutierrez, Rafael Barreto, Meijing Wang, David L. Waning, Lilian I. Plotkin and Andrea Bonetto

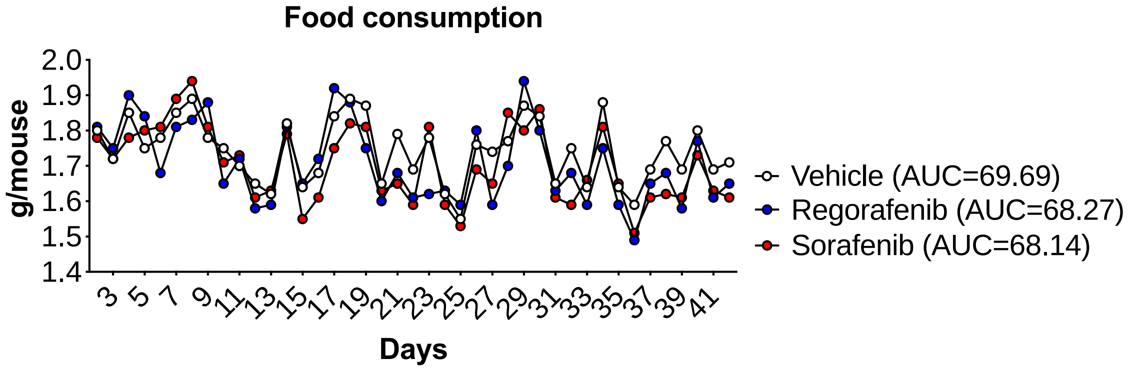

Figure S1. Food consumption is not affected by regorafenib or sorafenib. Weight of consumed food was measured in mice treated with 30 mg/kg/day regorafenib (blue; n = 8), 60 mg/kg/day sorafenib (red; n = 8), or vehicle (white; n = 8) over the course of 6 weeks. Each time point represents the average food intake, per mouse, in each experimental group. The area under the curve (AUC) for the three groups is also reported.

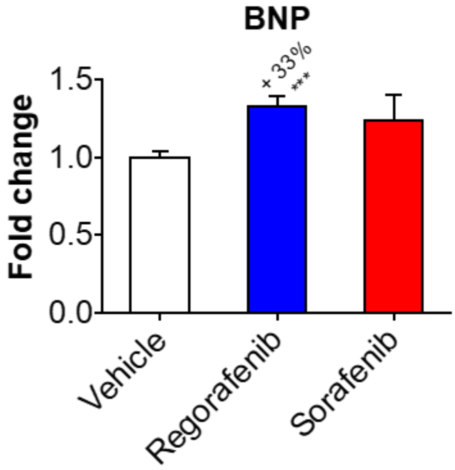

Figure S2. BNP mRNA expression is increased in animals treated with MKIs. mRNA expression for BNP was assessed in mice treated with 30 mg/kg/day regorafenib (blue; n = 8), 60 mg/kg/day sorafenib (red; n = 8), or vehicle (white; n = 8) over the course of 6 weeks. Data presented as mean ± SEM. Significance of the differences: *** $p < 0.001$ vs. Vehicle.

© 2019 by the authors. Licensee MDPI, Basel, Switzerland. This article is an open access article distributed under the terms and conditions of the Creative Commons Attribution (CC BY) license (http://creativecommons.org/licenses/by/4.0/).
